# The effect of Q-switched 1064-nm dymium-doped yttrium aluminum garnet laser on the skin barrier and collagen synthesis through miR-24-3p

**DOI:** 10.1007/s10103-020-03214-9

**Published:** 2021-01-05

**Authors:** Zhi Yang, Xiaoxia Duan, Xue Wang, Dongqing Li, Qi Xu, Shunli Xiang, Birun Guo, Li He

**Affiliations:** Department of Dermatology, The First Affiliated Hospital of Kunming Medical University, No. 295, Xichang Road, Kunming, 650032 Yunnan Province People’s Republic of China

**Keywords:** 1064-nm Q-switched neodymium-doped yttrium aluminum garnet laser, Collagen, Skin barrier, miR-24-3p

## Abstract

Due to the increase of the world’s population aging, how to restore youthfulness to the skin has attracted much attention. It is well known that collagen synthesis and changes in skin barrier play an important role in the process of skin aging. However, whether Q-switched 1064-nm Nd:YAG laser (1064-QSNYL) determines the involvement of miRNAs in skin collagen synthesis and skin barrier changes remains to be elucidated. Upstream miRNAs of p38 molecular pathway have been predicted by bioinformatic database and the relationship between miRNAs and p38 verified by dual-luciferase reporter gene and Western blotting. RT-qPCR analysis detected the expression of miR-24-3p and mRNA for collagen and skin barrier–related molecules, such as keratin 10 (K10), filaggrin, and Aquaporin 4 (APQ4), in mice back skin and in the keratinocyte cell line HaCaT. Western blotting and immunofluorescence (IF) have been used to detect collagen expression and to localize, as well as quantify K10, filaggrin, and APQ4, respectively. In this study, we show that p38 is the main target gene of miRNA-24-3p, and laser irradiation at 1.5 J/cm^2^ inhibits miR-24-3p expression. Irradiation treatment upregulates the moisture, elasticity, hydroxyproline, and superoxide dismutase content of mice skin, as well as inhibits trans-epidermal water loss. Irradiation also increases collagen, K10, filaggrin, and APQ4 in both mice skin and HaCaT cells. Interestingly, we found that miR-24-3p overexpression inhibits the effect of irradiation on collagen synthesis and skin barrier. We show for the first time that 1064-QSNYL promotes collagen synthesis and protective effects on skin barrier by downregulating miR-24-3p.

## Introduction

Skin aging is the most evident aging process of the body, the skin being the outermost organ. Skin aging is characterized by loose skin, thinning, increased wrinkles and pigmentation, enlarged pores, and telangiectasia [1]. Pathologically, skin aging manifests as decreased collagen content, thinning of the dermis, and reduced skin elasticity [2]. Skin aging not only hinders aesthetics but also increases the psychological burden of patients, and eventually induces various benign or malignant skin tumors [3]. Therefore, exploring safe, effective methods to prevent and improve skin aging had become one of the hotspots in the field of dermatology.

Traditional treatment methods for skin aging include drug treatment [4], mechanical abrasion [5], and chemical peeling [6]. These methods have certain therapeutic effects in the initial stage, but their employments are limited by their cumbersome operation and frequent infections after surgery. In recent years, the application of laser equipment has been considered as one of the more effective treatments for anti-aging and promoting skin rejuvenation. Q-switched 1064-nm dymium-doped yttrium aluminum garnet laser (1064-QSNYL) was the first laser used for non-exfoliative skin rejuvenation [7, 8]. As early as 1997, Goldberg and Whitworth found that 1064-QSNYL with a spot diameter of 7 mm and an energy intensity of 2 J/cm^2^ could significantly improve skin texture, elasticity, and facial wrinkles [9]. In addition, the main cause of skin aging was the abnormal expression of collagen, matrix metallopeptidase (MMP), and tissue inhibitor of metalloproteinases (TIMP) in the skin [10, 11]. Our previous research found that 1064-QSNYL regulated the expression level of collagen, TIMP, and MMP by activating ERK1/2 and P38MAPK signaling pathways, which was beneficial to the non-ablation of hairless mouse skin [12], and the best effect was when laser irradiation at 1.5 J/cm^2^ [13]. At the moment, the signaling pathways upstream of p38 MAPK and activated by 1064-QSNYL treatment are still unknown.

MicroRNAs (miRNAs) are a class of non-coding single-stranded RNA molecules with a length of about 22 nucleotides encoded by endogenous genes. MiRNAs are involved in post-transcriptional gene expression regulation in both animals and plants [14]. A large number of studies have shown that miRNAs play a pivotal role during skin aging. For example, Tang et al. [15] analyzed abnormal miRNA expression in aged skin and found that miR-302b-3p is abnormally highly expressed in aged skin and that it accelerates skin aging process by targeting JNK2. Xie et al. [15] showed an opposite correlation between the expression of miR-377 and DNMT1 in young and photoaged fibroblasts, as miR-377 promoted the senescence of fibroblasts by inhibiting the expression of DNMT1. So far, there are no studies on the effects of 1064-QSNYL on skin barrier and collagen synthesis through miRNAs. Starting from a bioinformatic observation that miR-24-3p can act upstream of p38 MAPK, in this study, we explore the effects of 1064-QSNYL on skin barrier and collagen synthesis, potentially mediated by molecular mechanisms involving miR-24-3p and p38 MAPK. Our findings provide the experimental basis for the application of 1064-QSNYL for the prevention of skin aging.

## Materials and methods

### Animals and laser irradiation treatment

Six-week-old male SKH-1 hairless mice (Shanghai Public Health Clinical College, China) weighing 20–30 g were housed in a controlled environment with a light/dark cycle of 12 h and given food and water ad libitum. SKH-1 hairless mice were randomly divided into NC group, irradiation group, atopic dermatitis (AD)-NC + irradiation group, and AD-miR-24-3p + irradiation group: no irradiation and adenovirus injection in the NC group; 1064-QSNYL irradiation and no adenovirus injection in the irradiated group; 1064-QSNYL irradiation and AD-NC injection in the AD-NC + irradiation group; and 1064-QSNYL irradiation and AD-miR-24-3p injection in the AD-NC + irradiation group. 1064-QSNYL irradiation (Medlite IV, Conbio, USA) conditions are as follows: fluence of 1.5 J/cm^2^, a spot size of 6 mm, and a pulse width of 6 ns, 10 Hz. The fluences were conformed to the uniform distribution. The energy was delivered with 10% overlap, and the treatments were conducted twice a week, lasting for 4 weeks. A dynamic cooling device (Cryogen; Candela) sprayed cryogen was used to cool the epidermis before and after the laser irradiation was performed. Adenovirus (Hanbio, China) injection conditions are as follows: each of the male SKH-1 hairless mice was injected intracutaneously with 1 × 10^9^ pfu AD-NC or AD-miR-24-3p. The experiment was approved by the ethics committee of Kunming Medical University.

### Cell culture and transfections

Human immortalized keratinocyte HaCaT cell line (ATCC, USA) was cultured in RPMI-1640 (Gibco, USA) medium and grown at 37 °C in a 5% CO_2_ incubator (Forma, USA) for culture. When the cell adhesion reaches 80–90%, it was seeded in a 6-well plate at a cell density of 3 × 10^5^/ml. Cell transfection groups are as follows: control group, irradiation/cell group, mimic-NC + irradiation group, and miR-24-3p mimic + irradiation group. The transfection method was operated according to instructions of Lipofectamine™2000 transfection Kit (Invitrogen, USA). After 24 h of transfection, 1064-QSNYL irradiation conditions are as follows: 1.5 J/cm^2^, 2-Hz frequency, 10 time of irradiation to treat HaCaT cells in each group for 24 h. Then, HaCaT cells from each group were collected for subsequent experiments.

### Skin moisture, elasticity, trans-epidermal water loss analyses

Skin moisture, elasticity, and trans-epidermal water loss (TEWL) were analyzed by the multi-probe skin test system MPA 580 (Courage + Khazaka, GER) immediately, 7 days, 14 days, 21 days, and 28 days after the last laser irradiation. Mice were anesthetized by intraperitoneal injection of chloral hydrate before the test. Test process was performed in a closed chamber, at a temperature range of 19.8 °C–21.4 °C and relative humidity of 45.8–49%.

### Hydroxyproline and superoxide dismutase assays

Hydroxyproline and superoxide dismutase (SOD) contents were measured in skin tissues of mice immediately, 7 days, 14 days, 21 days, and 28 days day after the last laser irradiation. For each assay, 100 mg of skin tissues was taken from 2 similar areas of the mouse back, cut into as small pieces as possible with sterile ophthalmic scissors, and put in a 15-ml centrifuge tube. Hydroxyproline measurement was performed using the hydroxyproline kit (Jiancheng, China), following the manufacturer’s instructions. SOD content was determined using a SOD kit (Solarbio, China) on supernatants obtained from skin tissues undergone homogenization with PBS and centrifugation at 4 °C.

### Dual-luciferase reporter gene assay

Three bioinformatic databases, PicTar (https://pictar.mdc-berlin.de/), TargetScan (http://www.targetscan.org/vert_72/), and miRMap (https://mirmap.ezlab.org/), predicted the putative miRNAs upstream of p38. The WT-p38-3′-UTR and MUT-p38-3′-UTR genomic regions were inserted upstream of luciferase gene in the pGLO-basic vector, and then co-transfected with miR-NC and miR-24-3p mimic into 293T cells for 8 h at 37 °C. After 48 h of transfection, luciferase activities were measured using the dual-luciferase reporter gene assay kit (Beyotime, China) and a microplate reader.

### Quantitative real-time PCR

Trizol reagent (Qiagen, USA) was used to extract total RNA from skin tissue samples and HaCaT cells according to the manufacturer’s instruction. RevertAid™ First Strand cDNA Synthesis Kit (Invitrogen, USA) was used to reverse transcribe total RNA into cDNA. MiRNA and mRNA analysis was performed by quantitative real-time PCR (RT-qPCR) and using SYBR® Premix Ex Taq™ Kit (Takara, Japan). RT-qPCR reaction system were as follows: 10 μl TB Green Premix Ex Taq II, 0.8 μl PCR forward and reverse primers, 0.4 μl ROX Reference Dye, 2 μl DNA template, and 6 μl H_2_O_2_. RT-qPCR reaction conditions: 95 °C for 5 min, 94 °C denaturation for 30 s, 60 °C annealing for 30 s, 35 cycles. The primer sequences are shown in Table 1. The experimental results were calculated using the 2^−ΔΔCt^ method.

**Table 1:** Primers used in RT-qPCR

Name	Forward primer (5′–3′)	Reverse primer (5′–3′)
miR-24-3p	CTCTGCCTCCCGTGCCTA	TGTTCCTGCTGAACTGAGCC
miR-128-3p	GTTGGATTCGGGGCCGTAG	AAGCAGCTGAAAAAGAGACCG
miR-6361	CTCTCCAGGAAGTGGTGGGAA	ATGGGGCTGAATACTGTTGGGT
miR-6369	AGTGTTGGTGAGTGTCTA	GGVTATCCTCTGCTACATA
miR-6410	CTCCTGGGGACCTTGTTTGG	CATGGAACTCCTCGGCCCTA
miR-6413	CTGAGCCATCTCTCCAGTGTC	TTGCCTGTCCTTGCCTAAGT
miR-6539	ACAGTGATGAACTCTGAGGGC	ACATTCACAGACCCAGTGAGC
miR-6540-5p	GCAAAGGCTCTCCTAAGGCA	AAGGCCCTTTGGAGGTAAGC
U6	CCCTTCGGGGACATCCGATA	TTTGTGCGTGTCATCCTTGC
P38	TGTGAACGAAGACTGTGA	TAGCCTGTCATCTCATCAT
Collagen I	AGGTATGCTTGATCTGTAT	CAGTCCAGTTCTTCATTG
Collagen III	TGAAGATGTCGTTGATGT	GCAGTGGTATGTAATGTTC
Collagen IV	GCATAGTCAGACAACAGAT	TTGGACATAGAGCAGAGA
K10	AGAGTGGTTCAATCAGAAGA	AGACTTATGGCTGGACATT
Filaggrin	ATGGTGGAACTGATGGAA	GAATCTTGTTGGTGTCTGT
APQ4	CCTTCTAAGCCACAATAG	AGCCTAACTCATCGTAAT
β-actin	TTCCAGCCTTCCTTCTTG	TGTCAACGTCACACTTCA

### Western blotting

Skin tissue samples and HaCaT cells were lysed using RIPA lysis buffer on ice, and total proteins quantified using BCA Kit (Beyotime, China). A total of 20 μg of protein samples was used for Western blotting analysis. The samples were subjected to SDS-PAGE (10%) at 200 V, 300 mA for 50 min, and blocked by 5% (*w/v*) dry milk in TBS for 1 h at room temperature. Membranes were incubated with the primary antibodies. Membranes were incubated at 4 °C overnight with the following primary antibodies: anti-p38 (1:1000, ABclonal Biotechnology, China); anti-Collagen I (1: 1000, Cell Signaling, USA); anti-Collagen III (1:1000, Proteintech, USA); anti-Collagen IV (1: 1000, Abcam, UK); and anti-β-actin (1:1000, Santa Cruz Biotechnology, USA) antibodies. Anti-rabbit secondary antibody conjugated to horseradish peroxidase (HRP) was used to visualize the stained bands with an enhanced chemiluminescence kit (Millipore, USA). Bio-Rad gel imaging system (Invitrogen, USA) was used to photograph bands. β-Actin was used as an internal control. Image J software was used to analyze the band intensity and the relative band intensity of each sample was normalized to β-actin signal in the same lane.

### Immunohistochemistry staining

Paraffin sections of skin tissue were dewaxed, re-hydrated, and incubated with 30 mL/LH_2_O_2_ for 10 min. After soaking the paraffin sections in PBS for 5 min, PBS containing 100 g/L BSA was added and then incubated for 10 min. Polyclonal rabbit anti-Cytokeratin 10 (keratin 10 (K10), Abcam, UK), polyclonal rabbit anti-filaggrin (Abcam, UK), and polyclonal rabbit anti-Aquaporin 4 (APQ4, Abcam, UK) were added to sections in a wet box at 4 °C. After 3 washes with PBS, sections were incubated for 30 min with HRP-labeled goat anti-rabbit IgG secondary antibody. After washing 3 times with PBS, DAB color development, hematlignin redyeing, and neutral gum sealing were performed and observed with a laser confocal microscope.

### Immunofluorescence staining

For immunofluorescence (IF) staining, HaCaT cells were fixed with 4% paraformaldehyde in PBS for 15 min at room temperature, permeabilized with 0.1% Triton X-100 for 10 min, and then blocked with 5% BSA for 1 h. Then, samples were incubated overnight at 4 °C with polyclonal rabbit anti-K10 (K10, 1: 150, Abcam, UK), polyclonal rabbit anti-filaggrin (1:100, Abcam, UK), and polyclonal rabbit anti-APQ4 (1:200, Abcam, UK) antibodies. After 3 washes with PBS, cells were incubated with goat anti-rabbit IgG H&L (1:500, Abcam, UK) at room temperature in the dark for 2 h. For nuclear counterstaining, samples were incubated with DAP (Sigma, USA) for 5 min at room temperature. Finally, immunofluorescence images were captured by using an inverted fluorescence microscope (Olympus, Tokyo, Japan). Image J software was used to measure the average absorbance and to analyze the results.

### Statistical analysis

Statistical analyses were performed with SPSS 18.0 software. All experiments were performed using eight different mice. Results were expressed as mean ± standard deviation (SD). Differences between groups were evaluated with Student’s *t* test. *P* < 0.05 was considered statistically significant. All data analyses were independently repeated three times.

## Results

### 1064-QSNYL upregulates the expression level of p38, whereas miR-24-3p targets and negatively regulates p38

**Fig. 1:**
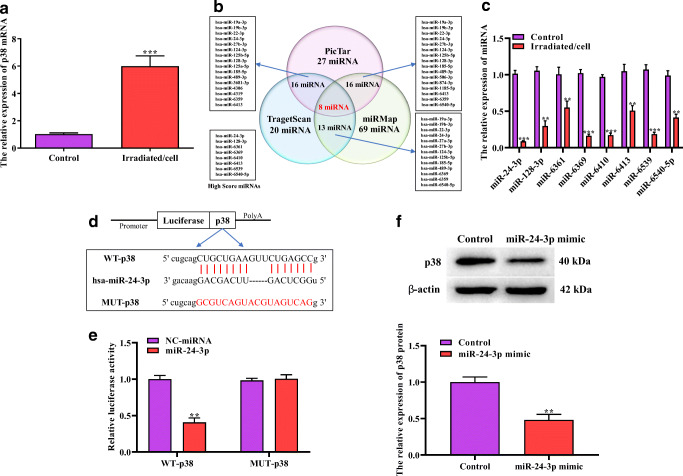
1064-QSNYL upregulated the expression level of p38, whereas miR-24-3p targeted and negatively regulated p38. **a** Effects of laser irradiation on the protein expressions of p38 were detected by RT-qPCR. **b** Venn diagram showing the miRNAs that target p38 in PicTar, TargetScan, and miRMap database. **c** Effects of laser irradiation on miRNAs that target p38 were detected by RT-qPCR. **d** Sequence alignment of the miR-24-3p base-pairing site in the 3′UTR of p38 mRNAs. **e**, **f** The targeting relationship of miR-24-3p and p38 was verified by dual-luciferase reporter gene and Western blotting. Data were expressed as mean ± SD. Comparison with control group, ^*^P < 0.05, ^**^P < 0.01, ^***^P < 0.001

In previous studies, we found that 1064-QSNYL irradiation promoted the skin collagen synthesis by activating the P38MAPK pathway, and the 1064-QSNYL irradiation at 1.5 J/cm^2^ had the best effect. In the present work, we used 1064-QSNYL irradiation at 1.5 J/cm^2^ to treat male SKH-1 hairless mice and found that it could upregulate the expression level of p38 mRNA (Fig. 1a), which was consistent with the previous experimental results. Furthermore, we found that eight miRNAs showed high scores with p38 by using three bioinformatics databases (Fig. 1b). We thus explored the effect of 1.5 J/cm^2^ 1064-QSNYL on the expression of these miRNAs by RT-qPCR. Interestingly, 1.5 J/cm^2^ 1064-QSNYL inhibited the expression levels of all miRNA, with a particular inhibitory effect on miR-24-3p (Fig. 1c). Later, we selected miR-24-3p to verify the targeting relationship between miR-24-3p and p38. The results of the dual-luciferase reporter gene showed that miR-24-3p could inhibit the luciferase activity of WT-p38 and had no significant inhibitory effect on the luciferase activity of MUT-p38 (Fig. 1d, e). In addition, the results of western blotting showed that overexpression of miR-24-3p significantly inhibited the expression level of p38 (Fig. 1f). Therefore, p38 is the target gene of miR-24-3p, and 1.5 J/cm^2^ 1064-QSNYL can inhibit the expression level of miR-24-3p.

### The effect of 1064-QSNYL irradiation on skin barrier in mice through miR-24-3p

**Fig. 2:**
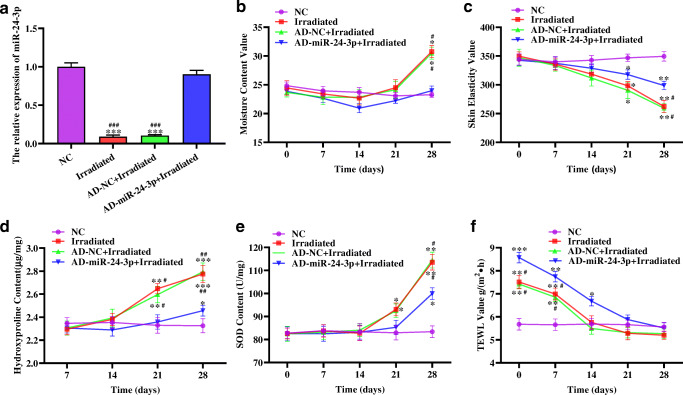
The effect of miR-24-3p on the skin barrier in mice after 1064-QSNYL irradiation. **a** RT-qPCR detected the expression level of miR-24-3p in each group. **b**, **c**, **f** Multi-probe skin test system detected moisture content, skin elasticity, and TEWL immediately and up to the 28th day. **d**, **e** Hydroxyproline and SOD assays detected hydroxyproline and SOD content, respectively, up to 28 days. Data were expressed as mean ± SD. Comparison with NC group, ^*^P < 0.05, ^**^P < 0.01, ^***^P < 0.001. Comparison with AD-miR-24-3p + irradiated group, ^#^*P* < 0.05, ^##^*P* < 0.01, ^###^*P* < 0.001

**Fig. 3:**
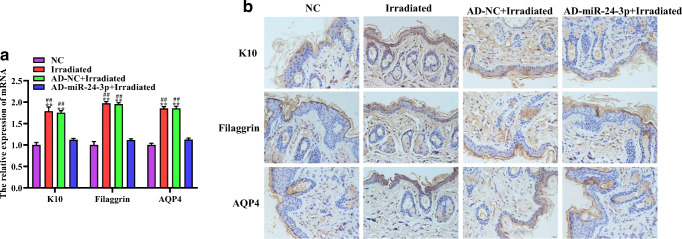
The effect of miR-24-3p on the skin barrier in mice after 1064-QSNYL irradiation. **a** RT-qPCR detected the expression level of K10, filaggrin, and AQP4. **b** Localization and quantification of K10, filaggrin, and AQP4 were performed by immunohistochemistry. Original magnification: × 40. Data were expressed as mean ± SD. Comparison with NC group, ^**^P < 0.01. Comparison with AD-miR-24-3p + irradiated group, ^##^*P* < 0.01

We measured moisture content, skin elasticity, hydroxyproline content, SOD content, and TEWL as evaluation indicators for the skin barrier integrity and function. RT-qPCR results showed that compared with the NC group, the expression level of miR-24-3p in the irradiated group and the AD-NC + irradiated group was significantly upregulated. The expression level of miR-24-3p in the AD-miR-24-3p + irradiated group was not significantly different from the NC group (Fig. 2a). In addition, on 28 days, moisture content, skin elasticity, hydroxyproline content, and SOD content in irradiated group and AD-NC + irradiated group were significantly higher than those in NC group and AD-miR-24-3p + irradiated group, of which all indicators in the AD-miR-24-3p + irradiated group were higher than those in the NC group (Fig. 2b–e). In addition, TEWL value in the irradiated group, AD-NC + irradiated group, and AD-miR-24-3p + irradiated group was significantly higher than that in the NC group from the beginning of irradiation. In the 14-day analysis, TEWL values in irradiated group and AD-NC + irradiated group were not significantly different from the NC group (Fig. 2f). Furthermore, we detected the expression levels of K10, filaggrin, and AQP4 related to the skin barrier. RT-qPCR and immunohistochemistry (IHC) results showed that the expression levels of K10, filaggrin, and AQP4 in the irradiated group and AD-NC + irradiated group were significantly higher than those in the NC group and AD-miR-24-3p + irradiated group, of which AD-miR-24-3p + irradiated group was higher than NC group (Fig. 3a, b). As a whole, these data indicate that 1064-QSNYL irradiation at 1.5 J/cm^2^ had a protective effect on the skin barrier and that overexpression of miR-24-3p could reverse this effect.

### The effect of 1064-QSNYL irradiation on collagen synthesis in mice through miR-24-3p

**Fig. 4:**
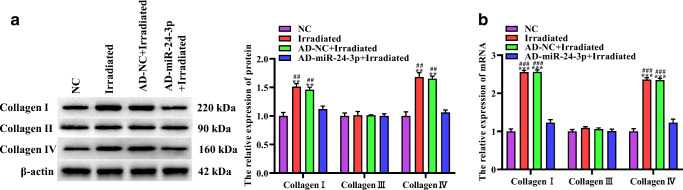
The effect of miR-24-3p on collagen synthesis in mice after 1064-QSNYL irradiation. **a**, **b** Protein and mRNA expression of collagen I, collagen III, and collagen IV were detected by Western blotting and RT-qPCR, respectively. Data were expressed as mean ± SD. Compared with NC group, ^**^P < 0.01, ^***^P < 0.001. Comparison with AD-miR-24-3p + irradiated group, ^##^*P* < 0.01, ^###^*P* < 0.001

To detect the expression of collagen, skin samples were taken after laser irradiation at 1.5 J/cm^2^ for 24 h, and Western blotting and RT-qPCR were performed. Western blotting results showed that the expression levels of collagen I and collagen IV were not significantly different in the irradiated group and the AD-NC + irradiated group, as well as in the NC group and the AD-miR-24-3p + irradiated group. The expression levels of collagen I and collagen IV of the irradiated group and AD-NC + irradiated group were higher than the NC group and AD-miR-24-3p + irradiated group. In addition, there is no significant difference in collagen III protein expression level was found in the four groups (Fig. 4a). Furthermore, RT-qPCR results showed that after 1064-QSNYL irradiation at 1.5 J/cm^2^, compared with the NC group and AD-miR-24-3p + irradiated group, the expression levels of collagen I and collagen IV mRNA in the irradiated group and AD-NC + irradiated group were significantly upregulated. On the contrary, the expression level of collagen III mRNA did not significantly change (Fig. 4b). Our data show that 1064-QSNYL irradiation at 1.5 J/cm^2^ promotes the collagen synthesis and that overexpression of miR-24-3p inhibits his process.

### The effect of 1064-QSNYL irradiation on collagen synthesis and skin barrier–related protein expression in HaCaT cells through miR-24-3p

**Fig. 5:**
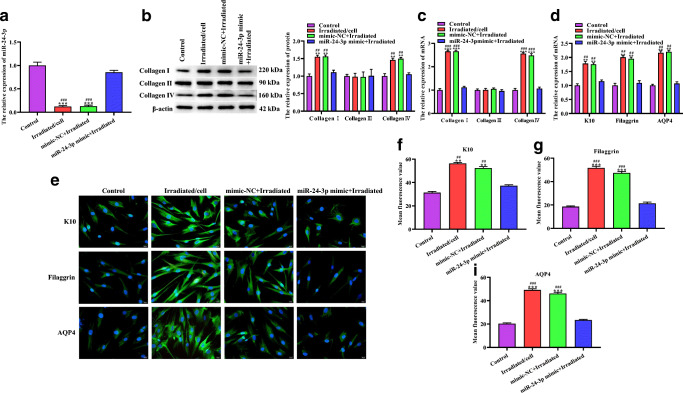
The effect of miR-24-3p on collagen synthesis and skin barrier–related protein expression in HaCaT cells after 1064-QSNYL irradiation. **a** RT-qPCR detected the expression level of miR-24-3p in each group. **b**, **c** Protein and mRNA expression of collagen I, collagen III, and collagen IV were detected by Western blotting and RT-qPCR, respectively. **d** RT-qPCR detected the mRNA expression level of K10, filaggrin, and AQP4 in each group. **e**, **f** Localization and quantification of K10, filaggrin, and AQP4 by Immunofluorescence. Original magnification: × 40. Data were expressed as mean ± SD. Comparison with the control group, ^**^P < 0.01, ^***^P < 0.001. Comparison with mimic-NC + irradiated group, ^##^*P* < 0.01, ^###^*P* < 0.001

We further explored the effect of overexpression of miR-24-3p on collagen synthesis and the expression level of the skin barrier–related proteins K10, filaggrin, and AQP4 in HaCaT cells after 1064-QSNYL irradiation at 1.5 J/cm^2^ at the cellular level. RT-qPCR results showed that after 1064-QSNYL irradiation at 1.5 J/cm^2^, the expression level of miR-24-3p was significantly downregulated, even though no significant difference in the expression level of miR-24-3p between mimic-NC + irradiated group and the control group was observed (Fig. 5a). Furthermore, we analyzed the expression of collagen in four groups of HaCaT cells by Western blotting and RT-qPCR. The results showed that compared with the control group and miR-24-3p mimic + irradiated group, the irradiated/cell group and mimic-NC + irradiated group showed significantly higher expression levels of collagen I and collagen IV, and there was no significant difference in the expression level of collagen I and collagen IV between control group and miR-24-3p mimic + irradiated group. Interestingly, collagen III did not vary in the four groups of HaCaT cells (Fig. 5b, c). We next analyzed the expression levels of skin barrier–related proteins K10 K10, filaggrin, and AQP4 in the four groups of HaCaT cells by RT-qPCR and IF. RT-qPCR results showed that the expression levels of K10, filaggrin, and AQP4 mRNA in the irradiated/cell group and mimic-NC + irradiated group were significantly higher than those observed in the control group and miR-24-3p mimic + irradiated group. No significant difference was found in the control group and miR-24-3p mimic + irradiated group (Fig. 5d). The IF results also showed that K10, filaggrin, and AQP4 are mainly localized in the cytoplasm. After 1064-QSNYL irradiation at 1.5 J/cm^2^, the average fluorescence values of K10, filaggrin, and AQP4 significantly increased. Importantly, after overexpression of miR-24-3p, the average fluorescence values of K10, filaggrin, and AQP4 were restored (Fig. 5e–f). Our data show that overexpression of miR-24-3p inhibits 1064-QSNYL irradiation at 1.5 J/cm^2^ on the induction of collagen synthesis and on the upregulation of skin barrier–related protein expression in HaCaT cells.

## Discussion

Skin aging is part of the aging of the human body. The combined action of internal and external factors in the human body leads to the destruction of the structural integrity of the skin and loss of physiological functions, which ultimately determine skin aging. With the development of science and technology, beauty equipment including microneedles [16], laser [17], radiofrequency, and ultrasound [18] have begun to be used in skin rejuvenation treatments, which enhanced the elasticity of the skin and increase water content to achieve the purpose of delaying skin aging. The application of laser equipment is considered one of the more effective methods to counteract skin aging and promote skin rejuvenation. 1064-QSNYL was the first laser used for non-exfoliative skin rejuvenation. It has the characteristics of strong penetrating power and very short pulse width; thus, a higher energy dose can be applied to the dermis without damaging the epidermis. The principle is that it easily penetrates the dermal layer of the skin to produce a thermal effect, which in turn causes the activation of dermal fibroblasts and induces damage and repair reactions, thereby promoting the synthesis and remodeling of new collagen in the skin [19]. The biggest advantage of 1064-QSNYL is that the healing time after treatment is short, the side effects are small, and the normal life of the patient is not affected. It is more suitable for mild and moderate skin wrinkles and general light damage [20]. Therefore, this technology had received great attention as soon as it appeared.

Previous findings showed that, when Q-switched 1064-nm lasers were operated without any cooling system, the photothermal damage produced could easily cause damage to the skin barrier and delay the onset of action [21]. The skin barrier is mainly composed of the stratum corneum and granular layer. Cytokeratin, filaggrin, and AQP4 in the stratum corneum are cross-linked to form a keratinized envelope, and the flattened keratin plaques are embedded in it to form the physical barrier of the skin [22]. K10 is the main component of type I intermediate filament proteins, which plays an important role in the process of epidermal cell differentiation and migration [23]. In addition, filaggrin is an important structural protein in the skin barrier, produced in the stratum corneum of the epidermis, and has the function of cross-linking keratin fibers [24]. Intermediate filament proteins can eventually be degraded into free amino acids to form a moisturizing complex that maintains skin moisture and elasticity [25]. TEWL is an important indicator for evaluating skin barrier function. A number of studies have confirmed that TEWL values and skin barrier integrity are inversely related [26, 27]. In addition, the increase of reactive oxygen species (ROS) in skin tissues is one of the main reasons for skin aging after ultraviolet radiation [28]. SOD is an antioxidant enzyme widely distributed in organisms that can block and repair the damage caused by oxygen free radicals to cells [29]. It is the primary substance for scavenging free radicals in organisms, and previous studies have reported that the increase in SOD activity in skin tissue can delay skin aging [30, 31]. Therefore, we used moisture content, skin elasticity, hydroxyproline content, SOD content, TEWL, and skin barrier–related protein (K10, filaggrin, and AQP4) as indicators of skin barrier integrity. We found that 1064-QSNYL irradiation at 1.5 J/cm^2^ could significantly upregulate moisture content, skin elasticity, hydroxyproline content, SOD content, and the expression level of skin barrier–related protein of mice, but overexpression of miR-24-3p could restore the level of these indicators. Interestingly, immediately after irradiation treatment, TEWL value of the back skin of the mice significantly increased, to return to the basal level after 14 days.

Collagen is an unbranched protein synthesized by fibroblasts, which accounts for about 1/3 of the total protein in mammals. Collagen is the main structural substance of the dermal layer of the skin, and changes in the structure of the dermal layer are the main cause of skin aging [32]. The balance of collagen synthesis and catabolism determines the speed of skin renewal and skin aging. Previous studies demonstrated that other than maintaining skin elasticity, collagen has a certain degree of hardness to protect the skin from external damage and can promote cell adhesion and proliferation. In addition, collagen plays an important role in the regeneration of the human epidermis and having a regulatory effect on keratinocytes in the epidermal layer [33]. We found that 1064-QSNYL could significantly upregulate the expression levels of collagen I and collagen IV in the back skin of mice, as well as in HaCaT cells. Laser irradiation also inhibited the expression of miR-24-3p, and vice versa overexpression miR-24-3p could inhibit the effect of Q-switched 1064-nm Nd: YAG laser. In this study, we discuss the miR-24-3p-mediated beneficial effect of 1064-QSNYL on skin barrier and collagen synthesis. Our findings demonstrated that 1064-QSNYL can inhibit the expression level of miR-24-3p, thereby promoting collagen I and collagen IV in mice and HaCaT cells and enhancing the skin barrier of mice. However, whether 1064-QSNYL regulates the expression of p38 through miR-24-3p, and the mechanism of its effect on the skin barrier and collagen synthesis, in mice remains to be elucidated.
